# A Datasheet for the Age-Related Eye Disease Study (AREDS) on the database of Genotypes and Phenotypes

**DOI:** 10.1016/j.xops.2026.101115

**Published:** 2026-02-11

**Authors:** Minali Prasad, Akanksha Nagarkar, Elvira Agron, Claire Weber, Emily Y. Chew, Tharindu De Silva, Souvick Mukherjee

**Affiliations:** Division of Epidemiology and Clinical Applications, National Eye Institute, National Institutes of Health, Bethesda, Maryland

**Keywords:** Cataract, Age-related macular degeneration, AREDS

## Abstract

**Objective:**

Over the years, the Age-Related Eye Disease Study (AREDS) has contributed to the natural history and understanding of progression related to age-related macular degeneration (AMD) and cataracts. This paper summarizes AREDS data elements available for research.

**Design:**

Data set description for data acquired during AREDS.

**Participants:**

Adults aged 55 to 80 years with no to advanced AMD in 1 eye but visual acuity >20/32 in eye without advanced AMD.

**Methods:**

database of Genotypes and Phenotypes archives and distributes data acquired in clinical studies such as AREDS. This article provides a detailed description of the data available under controlled access under the National Institutes of Health genomic data sharing Policy.

**Main Outcome Measures:**

Age-Related Eye Disease Study evaluated progression to advanced AMD, 15-letter decrease in visual acuity, and progression of lens opacities as primary outcome measures.

**Results:**

The data set includes 4757 participants enrolled across 11 participating clinical centers representing varying AMD severity categories and lens opacities. The participants' demographics and clinical variables were collected during baseline and follow-up visits, and data elements included ophthalmic images (fundus and lens photographs) with reading center gradings; nutritional estimates captured from food frequency questionnaires; data related to quality of life, hospitalization, morbidity, and mortality; and genetic data (available in a subset of approximately 2400 participants who submitted blood samples).

**Conclusions:**

Summary of data available under controlled access acquired as part of AREDS was provided. The AREDS data set offers a valuable resource for advancing our understanding of AMD and cataract progression and for developing novel tools and applications to transform ophthalmic diagnostics and therapeutics.

**Financial Disclosure(s):**

Proprietary or commercial disclosure may be found in the Footnotes and Disclosures at the end of this article.

Among individuals aged ≥50 years, the leading causes of blindness include age-related conditions such as macular degeneration and cataracts.^1^ Age-related macular degeneration (AMD) is responsible for 8.7% of blindness globally, and cataracts are commonly incident in the fifth and sixth decade of life.^2^^,^^3^ Given the increase in elderly populations worldwide, the Age-Related Eye Disease Study (AREDS) catered to the growing need to investigate the natural history and risk factors and to assist in the development of effective therapies for age-related eye diseases.

Prior to AREDS, there existed a few population-based cohort studies for AMD and cataracts. The most notable studies included the Framingham Eye Study,^4^ the Beaver Dam Eye Study,^5^ the Blue Mountains Eye Study,^6^ and the Rotterdam study.^7^ To further understand pathophysiology in age-related eye diseases, AREDS was designed as a prospective cohort study aimed to report the incidence, course, and progression of AMD and lens opacities as well as determine the risk factors associated with the development and progression of these conditions. Participants were recruited from 11 clinical centers from across the United States and followed a standardized protocol throughout the study.

Over the years, AREDS have contributed a plethora of knowledge in our understanding of AMD and cataract-related disease progression and associated risk factors. This data set has also helped develop disease severity scales and recommendations for nutritional supplementation. The study collected multimodal data, including demographic, clinical, imaging, genetic, and patient-reported outcomes. Given the recent advances in artificial intelligence (AI) and data science, the AREDS data has already enabled the development of deep learning models for AMD staging and progression.^8^^,^^9^ With rigorous external validation and integration of complementary data sets, such AI tools could enable earlier identification of high-risk patients and support efficient therapeutic interventions to slow disease progression. This imperative is reinforced by 2 recent prospective, multimodal-imaging cohorts: the HONU Study^10^ and the MACUSTAR Study,^11^ both of which demonstrate the power of advanced imaging biomarkers in intermediate AMD and underscore the need for earlier patient stratification.

This current report will hopefully further raise the awareness of these data which may help to transform disease understanding and improve patient care. Its rich, longitudinal repository of fundus images, visual function assessments, and clinical variables with reading-center gradings provide an ideal foundation for developing algorithms to enhance AMD staging and predict progression risk.^9^ While the AREDS study is well-known, the granular details of the data set have not been previously synthesized into a single resource. These details could be a valuable resource for data science researchers in ophthalmology to swiftly understand the structure and content and utilize the data set. In this paper, we outline the rationale, composition, acquisition methods, and distribution of the AREDS data set to empower researchers in data-intensive studies.

## Datasheet

As described earlier, AREDS was designed to characterize the natural history and progression of AMD and cataract, as well as identify associated risk factors with these conditions. Table 1 illustrates a summary of main findings of the study.^12^, ^13^, ^14^, ^15^, ^16^, ^17^

**Table 1:** Articles Describing the Main Findings of AREDS

Report No.	Study Groups	Main Findings	Significance
3 (2000)^12^	(1) Controls; (2) intermediate drusen; (3) large drusen; (4) geographic atrophy (GA); (5) neovascular AMD	**Risk factors associated with AMD:** smoking, hypertension, hyperopia, lens opacities, less education, female gender, increased body mass index, and White race were associated with AMD.	Analyzed the association of demographic, medical history, medication, and ocular based risk factors with AMD
4 (2001)^13^	(1) Nuclear opacities; (2) posterior subcapsular opacities; (3) cortical opacities	**AREDS lens opacities grading:** high reliability in grading lens opacities using the AREDS system for classifying cataracts from photographs.	Described a 7-step system developed for grading the AREDS participants for nuclear opacities from slit lamp photographs based on the 4-step Wisconsin system for grading cataracts from photographs.
8 (2001)^14^	(1) Antioxidants (vitamin C, 500 mg; vitamin E, 400 IU; and beta carotene, 15 mg); (2) zinc, 80 mg, as zinc oxide and copper, 2 mg, as cupric oxide; (3) antioxidants plus zinc; or (4) placebo	**Primary results for AMD:** antioxidants plus zinc significantly reduced the odds of advanced AMD development and moderate visual acuity loss.	Development of over-the-counter AREDS oral supplements containing vitamins C, E, beta-carotene, and zinc and copper
9 (2001)^15^	(1) No antioxidants: (a) placebo or (b) zinc, 80 mg, as zinc oxide and copper, 2 mg, as cupric oxide or (2) Antioxidants: (a) without zinc (vitamin C, 500 mg; vitamin E, 400 IU; beta carotene, 15 mg) or (b) with zinc (antioxidants with zinc, 80 mg, as zinc oxide and copper, 2 mg, as cupric oxide)	**Primary results for lens opacities:** there were no significant differences in primary outcomes increase in baseline in nuclear, cortical, or posterior subcapsular opacity grades or cataract surgery, and (2) ≥15 letters of visual acuity loss between study groups.	High-dose antioxidant supplementation had no significant impact on the development or progression of lens opacities and associated visual acuity loss over a 7-year follow-up period.
17 (2005)^16^	Drusen area groups (μm represent radius): (1) none; (2) questionable; (3) small (<63 μm); (4) intermediate (63-124 μm); (5) large (125-249 μm); (6) very large (≥250 μm); Depigmentation area groups: (1) none; (2) questionable; (3) <0.056 disc area (DA); (4) ≥0.056 DA, <0.19 DA; (5) ≥0.19 DA, <0.5 DA, (6) ≥0.5 DA	**Development of the AREDS AMD classification**: the 5-year risk of advanced AMD increased from <1% in step 1 to ∼50% in step 9. Agreement of 87% of eyes between repeated gradings. The advanced stages of AMD are beyond the 9 steps.	Developed a 9-step severity scale using a 6-step drusen area scale with a 5-step pigmentary abnormality/noncentral geographic atrophy (GA) scale. Step 9: noncentral GA, step 10: central GA, step 11, neovascular AMD, step 12, both neovascular AMD and GA
18 (2005)^17^	4710 AREDS participants stratified by a simplified risk score based on the presence of ≥1 large drusen (≥125 μm) and any pigment abnormality in each eye.	**Development of the simplified AMD severity scale:** the 5-year risk of developing advanced AMD increases as the number of risk factors increase.	Developed the “simple scale”, a 5-step severity scale based on the presence of ≥1 large (125 μm) drusen and pigment abnormality in each eye.

AMD = age-related macular degeneration; AREDS = Age-Related Eye Disease Study.

### Data Set Composition

#### Overall Study, Data Sets, and Data Acquisition

The data set includes 4757 participants enrolled across 11 participating clinical centers representing several AMD severity categories (including no AMD) and cataract types (including no cataract). The data were acquired during the period 1992 to 2005 (1992–2001 randomized clinical trial, 2001–2005 epidemiologic follow-up study with every participant offered the AREDS supplement). Participants were enrolled based on fundus photograph gradings, best corrected visual acuity, and ophthalmic evaluation, and they were followed for a median time of 6.5 years and a maximum of 10 years.

**Figure 1:**
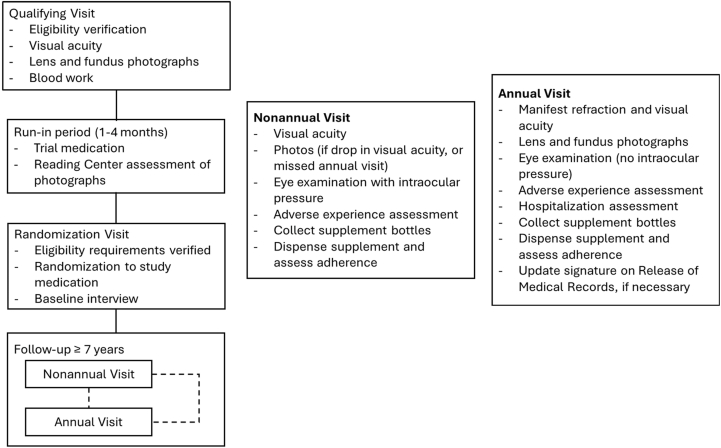
Summary of study randomization and protocol.

The 4 treatments groups were antioxidant supplementation (500 mg vitamin C, 400 mg vitamin E, 15 mg beta-carotene), zinc (80 mg zinc oxide) and copper (2 mg cupric oxide) supplementation, combined antioxidant and zinc supplementation, and placebo. The primary outcomes of the study were the progression to advanced AMD, 15-letter decrease in visual acuity score by the 3 treatment groups, comparing to placebo, and progression of lens opacities.^18^ The secondary analyses included evaluating the 2 outcomes of progression to advanced AMD and visual acuity decrease by comparing the “main effects,” between antioxidants vs. no antioxidants and between zinc vs. no zinc.^18^ Participants of the lens opacities portion of the clinical trial were randomized to antioxidant vitamins or placebo only if they did not have AMD.^18^ Follow-up visits were conducted every 6 months after randomization, annually during the natural history follow-up, and via phone between visits. The following information was collected during yearly in-person visits: ophthalmic procedures and comorbidities, visual acuity, intraocular pressure, lens and fundus examinations (excluding year 1), general medical information, use of prescribed and over-the-counter drugs, and other supplementation. The imaging was conducted at annual visits starting at the second annual visit and only at 6-month visits if the participant had visual acuity loss of ≥10 letters. Blood samples were collected from a subset of participants for genetic testing and were also evaluated for hemoglobin (to assess copper-deficiency anemia), cholesterol, and vitamin and copper levels according to AREDS Report No. 8.^14^ The summary of the study randomization and protocol is shown in Figure 1 and variables available are listed in Table 2.

**Table 2:** Summary of Data Available in AREDS with dbGaP Tables

Variables	∼N	Description	dbGaP Table Name (N of Variables)
Participants’ demographics and clinical data	297	Includes AREDS treatment assignment, sociodemographic characteristics, and baseline medical history. Medical history information includes eye disease information such as AMD category, visual acuity, and past ophthalmic treatments (e.g., cataract surgery, laser treatment) as well as information about the presence/absence of other diseases (e.g., angina, arthritis, cancer, diabetes, high blood pressure), body mass index, blood pressure, use of prescribed drugs for disease management (e.g., blood-thinning medications, digoxin, thyroid hormones, insulin), use of over-the-counter drugs (e.g., NSAIDs, antacids), intake of vitamins and minerals, and smoking history.	*genspecphenotype (116)* *enrollment-_randomization (101) followup (80)*
AMD/fundus-related data	88	Includes presence, type, and extent of drusen, presence/extent of RPE depigmentation, serous sensory retinal detachments, subretinal hemorrhages, subretinal fibrosis, and geographic atrophy. In addition, information on photocoagulation treatment for AMD SRNV is included, as well as AMD severity scale scores and AMD simple scale scores calculated from the fundus data.	*fundus (47)* *genspecfunduslens (41)*
Cataract/lens-related data	80	Includes cataract phenotypes and gradings related to bilateral nuclear, cortical, and posterior subcapsular opacity information included at baseline and end of study.	*amdlensphenotype (20)* *lens (19)* *genspecfunduslens (19) genspecphenotype(22)*
Dietary and supplement intake data	54	Includes information (n = 54 variables in table *dietary*) about AREDS participants' dietary intake of alcohol, amino acids, carbohydrates, fiber, poly-, and monounsaturated fatty acids, proteins, vitamins, and minerals. Includes glycemic index and glycemic load.	*dietary (54)*
Other data	79	Adverse events including type of adverse event, severity, primary ICD-9 code, outcome, potential relationship to study drug, timing, and treatment. Hospitalization information including reason, time from randomization, duration, primary ICD-9 code) during the entire study. Mortality information including cause and time from randomization. Information about participants’ use (e.g., type, frequency, dose, duration) of NSAIDs (e.g., aspirin, ibuprofen, acetaminophen, naproxen). NEI VFQ-25 evaluated participants’ general vision status and effect on behavior. The sunlight exposure variable (in table *sunlight*) describes participants’ annual exposure to sunlight in hours between April to September.	*adverse (11)* *hospitalization (5)* *mortality (6)* *nsaids (42)* *vfq (14)* *sunlight (1)*

AMD = age-related macular degeneration; AREDS = Age-Related Eye Disease Study; dbGaP = database of Genotypes and Phenotypes; ICD-9 = International Classification of Diseases, Ninth Revision; NEI = National Eye Institute; NSAIDs = nonsteroidal anti-inflammatory drugs; RPE = retinal pigment epithelium; SRNV = subretinal neovascularization; VFQ-25 = Visual Function Questionnaire-25.

#### Demographics and Clinical Variables

**Figure 2:**
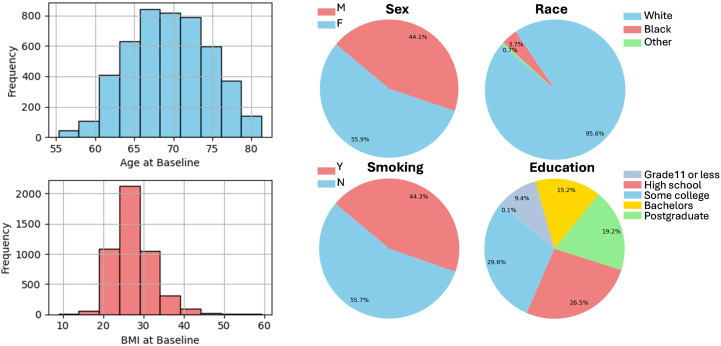
Sample patient demographics data available in AREDS. AREDS = Age-Related Eye Disease Study; BMI = body mass index.

Participants' demographics' and clinical characteristics data were well-characterized and included AMD and cataract phenotype information, medical history, sociodemographic information, other disease information, medical information, and smoking status. The participants population had age 69.4 ± 5.1 (years, mean [±standard deviation]), body mass index 27.5 ± 4.9 kg/m^2^, and was 56% female and predominantly of White race as illustrated in Figure 2.

#### Imaging Data

**Figure 3:**
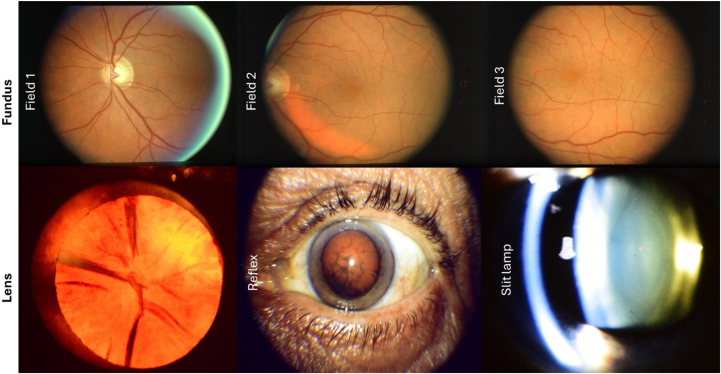
Example fundus and lens photographs available with the data set. Top: sample field 1, 2, and 3 color fundus photography, bottom: ample anterior Neitz (cortical opacity 98.5% per reading center), red reflex image, slit lamp images.

The data are accompanied by >134 500 fundus and lens photographs (Figure 3). The 30^o^, bilateral fundus photographs of fields 1, 2, and 3 were digitized and embedded in Digital Imaging and Communications in Medicine (DICOM) format. Images consisted of multiple longitudinal visits with an average follow-up of 8.9 ± 3.3 years with maximum of 13 years. The mean (±standard deviation) time between visits across all the participants was 481 (±194) days. The gradings for the photographs for both AMD and lens opacity were performed by a reading center and is available with the data.

#### AMD-Related Image Gradings

**Figure 4:**
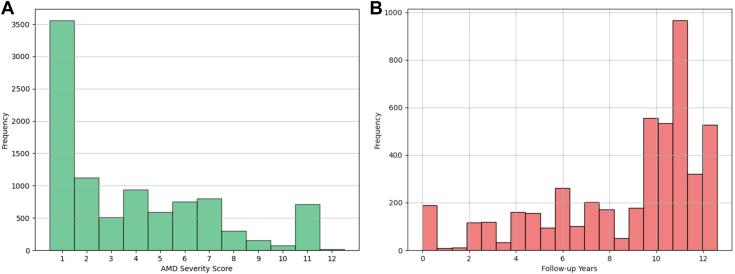
**A,** Distribution of AMD severity scores and **(B)** AMD gradings available at follow up years. AMD = age-related macular degeneration.

Graded AMD related image features included the presence or absence of drusenoid pigment epithelial defects, nondrusenoid pigment epithelial defects, serous sensory retinal/hemorrhagic retinal detachments, hard exudates, subretinal/sub-retinal pigment epithelium (RPE) hemorrhages, subretinal fibrosis, photocoagulation, AMD, geographic atrophy (GA), RPE depigmentation, increased pigmentation, confounding lesions, hard and soft drusen, reticular drusen, and calcified drusen. Investigators also recorded the maximum drusen size and area of RPE depigmentation, increased pigment, and GA area. Age-related macular degeneration severity scale scores were obtained in a 9-step scale^16^ with grading the presence of central GA (score 10), neovascular AMD (nAMD) (score 11), and central GA with nAMD (score 12) as separate categories. Figure 4 shows the distribution of AMD severity scale scores of the participants along with the longitudinal follow up available in terms of image gradings.

#### Lens Image Gradings

Anterior segment and lens gradings included bilateral nuclear, cortical, and subcapsular opacity information at baseline and longitudinal visits. Information related to iris pigmentation, nuclear color, and nuclear sclerosis for each eye was also recorded. Table 3 shows the distribution of nuclear opacity, posterior subcapsular opacity, and cortical opacity gradings data at baseline.

**Table 3:** Baseline Lens Opacity Gradings among Eyes of Participants Enrolled in the AREDS Study

	No. (%) (N = 9514 Eyes)
Baseline nuclear opacity score	
<2.0	1476 (15.5)
2.0–3.9	5544 (58.3)
≥4.0	2061 (21.7)
Not measured	433 (4.6)
Baseline cortical opacity within central 5-mm area, % of pupillary area	
<0.1	6079 (63.9)
0.1–4.9	2710 (28.5)
≥5.0	725 (7.6)
Baseline posterior subcapsular opacity within central 5-mm area, % of pupillary area	
<0.1	8937 (93.9)
0.1–4.9	445 (4.7)
≥5.0	132 (1.4)

AREDS = Age-Related Eye Disease Study.

#### Nutritional Data

The food frequency questionnaire was used to collect information about food intake, including questions regarding the annual frequency and serving size of various fruits and fruit-based products, breakfast foods, vegetables, meat, fish, poultry, lunch items, breads, snacks, and spreads. Participants' glycemic index and glycemic load were measured as part of the study. Based on the food frequency questionnaire, for each person, the nutrients variables were created by methods described in SanGiovanni et al.^19^

#### Genetics Data

Genetics samples were collected from approximately 2400 AREDS participants and analyzed with a modified HumanCoreExome array by Ilumina. Random sequencing of the whole genome was performed using blood specimens. Each participant's genotype for each single nucleotide polymorphism was recorded as wild type with no alterations, variation using standard genetic nomenclature, or no result. Image-based gradings and other data for these participants were linked. The genetic data were evaluated in Fritsche et al^20^ to identify common and rare variants of AMD and included as part of the International AMD Genetics Consortium. Currently, genetics data are available under database of Genotypes and Phenotypes accession numbers phs000001.v3.p1 and phs000429.v1.p1. The full whole-genome sequencing data set is currently being uploaded to database of Genotypes and Phenotypes and will be released under accession phs002494.v1.p1.

#### Quality of Life and Other Data

Other questionnaires administered during the study included the sunlight exposure questionnaire and the National Eye Institute (NEI) Visual Functioning Questionnaire-25 (VFQ-25). The sunlight questionnaire was used to assess the amount of sunlight exposure between April through September between 10 am and 4 pm based on the participants' location, daytime activities or jobs, and use of hats with a brim, sunglasses, and glasses or contact lenses. The VFQ-25 was administered to determine participants' beliefs and emotions about their visual conditions using the Likert-scale, defined as an attitude scale.^21^ Participants were asked about their overall health and ocular pain, difficulty with near and distant activities, social activities, driving, and mental health. Additionally, participants were asked about their use (type, frequency, dose, duration) of nonsteroidal anti-inflammatory drugs (e.g., aspirin, ibuprofen, acetaminophen, naproxen). Hospitalization, adverse events, and death were tracked during the study period. The data were adjudicated by committees for the final diagnosis associated with morbidity and mortality.

### Collection Process

The data collection involved 11 clinical centers nationwide using standardized protocols. The details of data collection are available in original study design papers and other technical documentation, as well as outlined here.^18^^,^^22^^,^^23^ To ensure high intergrader reliability and data validity, the study utilized a multitiered quality control framework. All clinical staff, including photographers, examiners, and technicians, were required to achieve certifications through standardized processes involving protocol trainings, written examinations, and the satisfactory submission of test data (e.g., refraction examinations or fundus images). Equipment adhered to strict specifications to ensure longitudinal consistency. Data entry utilized the AREDS Interactive Data Entry System, which restricted access to certified personnel and enforced logical constraints (e.g., forcing out-of-range value checks) at the point of entry. Specialized data, such as the NEI VFQ-25 and nutritional surveys, underwent centralized processing and real-time validation (e.g., same-day resolution of ambiguous items via the Minnesota Nutrition Data System) to minimize entry errors.

#### Ocular and Medical History, Physical Examination, and Surveys

Baseline interviews with multiple-choice questions were used to collect information about ocular and medical history. Participant height, weight, and blood pressure was recorded using standard methods. Participants were required to complete the food frequency questionnaire which was reviewed by the clinic coordinator for quality control. The 24-hour dietary recall was conducted over the phone by the Nutrition Coordinating Center at the University of Minnesota using a standardized telephone script. Responses were entered directly into the Minnesota Nutrition Data System, reviewed with the participant, and missing or ambiguous items were resolved via same-day calls. Participants also completed the sunlight exposure questionnaire during a follow-up visit. The NEI VFQ-25 was administered by trained interviewers and original questionnaires were mailed to the Coordinating Center for centralized processing.

#### Ophthalmic Examination

The participant underwent a comprehensive ophthalmic examination including slit lamp examination, ophthalmoscopy, intraocular pressure, and visual functional testing. At the qualifying visit, patients completed the visual acuity examination with their correction glasses, while at the nonannual visit, patients completed the visual acuity examination with the manifest refraction from the prior visit. The manifest refraction and visual acuity examination were conducted without dilation using standardized charts at fixed 4-m and 1-m testing distances under controlled room illumination using visual acuity light boxes. During testing, room lights were turned off so that the box provided illumination with 2 General Electric Cool Daylight 20-W fluorescent tubes. Each center maintained schedules documenting fluorescent tube conditioning, annual tube replacement, and periodic checks of tube function. Illumination at the chart surface was monitored to remain within the prespecified limit of ≤15 foot-candles, with tube status and lane setup reviewed during protocol monitoring visits. Intraocular pressure was measured with applanation tonometry, with pneumotonometer used if the former was not available. The ophthalmologist also completed a dilated fundus examination, particularly of the macula with direct ophthalmoscopy, slit lamp biomicroscopy, or both.

#### Imaging

Imaging was completed after dilating pupils to ≥5 mm for the qualifying visit (and ideally 6 mm for subsequent visits) and prior to a contact lens examination. Fundus and red reflex photographs were taken with a Zeiss FF-series camera using either a Kodachrome (ASA 25 and ASA 64) or Ektachrome (ASA 64 and ASA 100) film type. Fundus photographs were acquired at the following fields: (1) centered on the temporal edge of the optic disc; (2) one-eighth to one-quarter disc diameter above center of macula; and (3) three-quarters to 1 disc diameter temporal to center of macula. Red reflex photographs were acquired while focused on lens opacities if present or the pupillary margin of the iris if absent. The photograph was magnified such that the cornea is ∼13 mm on the film to allow for detection of lens opacities.

Lens photographs were acquired with the Topcon SL-6E Photo Slit Lamp camera (for slit lamp images) and the Neitz CT-R Cataract camera (for retro-illumination images). Both cameras were modified to increase reproducibility of images among clinical centers, among participants at each center, and between visits of the same participant. The Neitz camera was modified to control fixation and focus the photograph at a standardized depth within the lens. The Topcon camera was modified to standardize slit beam dimensions and control fixation. The slit lamp beam was standardized to 0.3 mm in width and 9.0 mm in height and locked at a 45-degree angle at the photographer's left with a 16X magnification. The cataract camera was used to acquire photographs at the edge of the pupillary margin and between 3 mm and 5 mm deep to the pupillary margin. Anterior and posterior images were captured due to the shallow depth of field of the Neitz camera. If a posterior subcapsular cataract was present, a third Neitz photograph was acquired while focusing on the cataract. Standardized maintenance protocols on a daily, weekly, annually, and as needed basis were implemented to ensure image quality.

In addition to the imaging certification process, there was a quality review program at the Reading Center to provide continuous feedback to photographers. If a fully certified photographer's image quality fell below the threshold for full certification over time (≥75% of ≥20 fundus or lens images graded as good or fair by the Reading Center), certification was reduced to provisional status and the photographer was provided with additional help to improve image quality. In the case of low fundus image quality in the absence of media opacities, the Reading Center requested retakes of the eye before determining eligibility for enrollment.

#### Blood Test

Fasting blood specimens (serum) were collected from 4 clinical centers (the Johns Hopkins Medical Institutions, Devers Eye Institute, National Eye Institute Clinical Center, and the Associated Retinal Consults) at baseline to measure levels of hematocrit high-density lipoprotein; low-density lipoprotein; total cholesterol; triglycerides; and vitamins A, E, and C; alpha- and beta-carotene; zinc; copper; lutein; zeaxanthin; β-cryptoxanthin; and lycopene.^14^

#### Genetic Testing

The Illumina Sentrix Human-1 Genotyping Beadchip platform was used by the Center for Inherited Disease Research to perform the genotyping.

### Screening Procedures

The qualifying visit was used to determine the eligibility of potential participants. This included an interview for medical history, visual acuity, lens assessment, and retinal assessment. Appendix A (available at www.ophthalmologyscience.org) lists eligibility criteria.

### Ethical Approval

The study procedures were formally approved by the AREDS operations committee, AREDS executive committee, the data and safety monitoring committee, and the institutional review board at the NEI. The institutional review board of each clinical center also approved the informed consent forms. Written consent was obtained from all participants during enrollment and participants were able to withdraw at any point during the study. The study adhered to the Declaration of Helsinki.

### Distribution of Data through database of Genotypes and Phenotypes

The AREDS data are available to investigators through the database of Genotypes and Phenotypes, a National Institutes of Health–designated controlled-access data repository under the National Institutes of Health genomic data sharing policy (accession number phs000001.v3.p1).^23^ Documentation required for the application includes a research use statement with objectives, study design, and an analysis plan, consistency with data use limitations, and plans for collaborations with other institutions. Applicants are also required to provide institutional and contact information of the study team. They are also required to provide the contact information for the IT Director, cloud use statement, and cloud servicer provider information (if applicable) to ensure guidelines related to data security and privacy. The research use statement is reviewed by a data access committee prior to providing data access.

### Application of the AREDS Data Set in Literature

Numerous investigators have already leveraged AREDS data to develop AI-assisted tools, novel applications, and provide additional disease insights (Table 4). Bhuiyan et al^8^ trained a convolutional neural network on nearly 120 000 AREDS color fundus photographs, achieving 99.2% accuracy for distinguishing intermediate/late versus early/no AMD and 86.4% accuracy for predicting 2-year incident late AMD. Burlina et al^9^ applied deep convolutional neural networks to grade AMD severity and estimate 5-year progression risk, reporting predictive errors of only 3.5% to 5.3%.

**Table 4:** Selected Publications Utilizing the AREDS Data Set

Study	Objective	Findings
Bhuiyan et al^8^	Develop an AI model for predicting progression to late dry and wet AMD.	Deep learning model demonstrated a 99.2% accuracy in discriminating between early/none and intermediate/late AMD. The model had a 66.88% accuracy for predicting late dry and 67.15% for late wet AMD.
Burlina et al^9^	Develop a deep learning model for estimating 5-year risk for progression to advanced AMD.	The model demonstrated mean estimation errors ranging from 3.47% to 5.29% for the 5-year risk.
Merle et al^23^	Evaluate the association between a Mediterranean diet and 10 genetic loci with progression to advanced AMD.	High adherence to a Mediterranean diet was significantly associated with lowered risk of progression in a multivariable model adjusted for genetic covariates.
Toulouie et al^24^	Examine the association between retinal vascular parameters and AMD severity or progression.	A lower arteriole-to-venule ratio, but not central retinal artery equivalent or central retinal vein equivalent, was associated with AMD severity. Choroidal neovascularization was associated with a narrower central retinal artery equivalent and arteriole-to-venule ratio. There were no vascular parameters associated with AMD progression.
Seddon et al^25^	Evaluate the relationship between genetic, ophthalmic, and environmental factors and AMD incidence and prevalence.	Five genetic variants (CFH Y402H, CFH rs1410996, LOC387715 A69S [ARMS2], C2 E318D, and C3 R102G) were associated with progression to advanced AMD. Smoking was also significantly associated with AMD risk.
Fritsche et al^26^	Identify genetic loci potentially involved in AMD pathophysiology.	COL8A1-FILIP1L, IER3-DDR1, SLC16A8, TGFBR1, RAD51B, ADAMTS9, and B3GALTL were associated with AMD cases.

AI = artificial intelligence; AMD = age-related macular degeneration; AREDS = Age-Related Eye Disease Study; CFH = complement factor H; SLC16A8 = solute carrier family 16 member 8.

Beyond AI applications, the AREDS data set has the potential to be used in future epidemiological and genetic studies. Many previous epidemiological studies have already demonstrated its utility of AREDS data. For example, Merle et al^24^ reported that following a Mediterranean diet was associated with decreased progression to advanced AMD. Toulouie et al found that a lower arteriole-to-venule ratio was associated with GA using fundus photos.^25^ Seddon et al^26^ combined genetic polymorphisms, baseline AMD grade, and lifestyle factors in a multimodal predictive model to detect advanced AMD prevalence and incidence. Fritsche et al^20^^,^^27^ used genome-wide association approaches in the AREDS cohort to identify 7 loci implicated in complement activation, lipid metabolism, extracellular matrix remodeling, and angiogenesis and subsequently reported a matrix metallopeptidase 9 variant associated with nAMD but not GA as well as rare variants in complement factor H, complement factor I, TIMP metallopeptidase inhibitor 3, and a splice variant in solute carrier family 16 member 8. Together, these studies illustrate the power of the AREDS data to drive AI-based diagnostics, risk modeling, genetic discovery, and biomarker development, with significant implications for precision medicine in AMD and cataract management.

### Strengths and Limitations

The strengths of the data set lie in the prospective nature, large sample size of participants, high retention rate of the participants, and extended study period designed to investigate several research questions. There is a proposal to prospectively study the use of AREDS2 supplements for the treatment of GA in a randomized clinical trial. The 2 clinical trials for AMD (Report 8) and cataracts (Report 9) were double-masked, and some participants with combined ocular findings were included in the analyses of one or both trials.^18^ The implementation of rigorous protocols for data acquisition, coupled with meticulous documentation, provides AREDS data set to be a valuable resource with high data quality with longitudinal ocular imaging and ophthalmic examinations.

However, some design aspects were also limitations of the study. Given the study eligibility criteria (Appendix A), participants who are volunteers tended to be healthier than the general population of the United States, which reduces the generalizability of the results.^14^ Also, the study population is predominantly White. While this limits the generalizability of findings to other racial and ethnic groups, it is important to note that this demographic skew mirrors the epidemiological disease burden; both advanced AMD and self-reported cataract surgery rates have been historically documented as more frequent among White individuals compared to other racial groups.^28^, ^29^, ^30^ Consequently, models trained solely on this data may underperform when applied to underrepresented populations, necessitating external validation on diverse data sets. Furthermore, the median age of participants was 68 years, implying that cataracts that were not clinically detectable may already have started forming by the time of antioxidant supplementation initiation.^15^ Retinal findings were based on color fundus photography only without fluorescein angiography which may have delayed identification of advanced AMD and underestimated the incidence of GA or nAMD.^14^ Other multimodal technologies for ophthalmic imaging that allow for earlier identification of AMD, including OCT, were not yet available. The exclusive use of fundus photography distinguishes this data set from multimodal OCT-based environments. Consequently, while direct application to OCT workflows is limited, the data set provides a rigorous standard for fundus-based analysis, which remains critical for disease diagnosis and longitudinal monitoring.

The study was unable to establish the optimal length of time that participants with intermediate AMD should use combination supplements, and participants in all study groups were found to progress to advanced AMD and moderate visual acuity loss.^14^ Detection of changes in lens opacities was confounded by changes in film, film development, and wear and tear of the lens imaging equipment.^15^ In data science–related projects, there could be generalizability limitations due to the past protocols for imaging and devices have evolved compared to what is currently available in clinical practice.

## Conclusions

The AREDS data set represents an unparalleled resource for ophthalmology and AI-driven clinical research in the context of a global aging population. The data have comprehensive multimodal imaging, including thousands of high-resolution color fundus photographs and lens images collected at baseline and annual follow-up visits of participants with and without AMD and cataracts. With the inclusion of patient-reported outcomes using the NEI-VFQ, investigators can study underlying relationships between structure, function, and lived experiences of participants with cataracts, AMD, or both. The set is longitudinal, with up to 10 years of follow-up information, including an interventional phase from 1992-2001, followed by an observational phase through 2005. By combining breadth, depth, and accessibility, the AREDS data stand to drive innovation in both algorithmic research and clinical decision support, paving the way for earlier diagnosis, personalized risk prediction, and more effective interventions in AMD and other age-related ocular diseases.

The AREDS data set should be used to explore further epidemiological, clinical, and AI-based research questions. Analyses should specify usage of the randomized treatment period, observational follow-up period, or both and outcome definitions should be aligned with established AREDS grading systems and endpoints. The longitudinal nature of the data warrants censoring from death or surgery as well as time-varying exposures such as supplement use and smoking. Authors should practice clear reporting of inclusion and exclusion criteria and study design choices.

Promising future directions for studies based on the AREDS data set include building and validating risk models trained on imaging, clinical, nutritional, and genetic information. Epidemiological studies could evaluate how combinations of genetic susceptibility, diet, and lifestyle influence progression to various AMD phenotypes, cataract surgery, and visual acuity loss. Studies could also examine how structural changes on fundus and lens photographs correlate with visual function and quality of life. By utilizing the multimodal data available, investigators can continue to address questions that influence the counseling and management of patients with AMD and cataracts.

## Data Availability

The information in this manuscript can be found in the database of Genotypes and Phenotypes (study accession: phs000001.v3.p1). This can be accessed with a formal application submission and review process (https://dbgap.ncbi.nlm.nih.gov/home/).

## References

[1] (2021). Causes of blindness and vision impairment in 2020 and trends over 30 years, and prevalence of avoidable blindness in relation to VISION 2020: the right to sight: an analysis for the Global Burden of Disease Study. Lancet Glob Health.

[2] Ruia S., Kaufman E.J. (2025). StatPearls.

[3] Nizami A.A., Gurnani B., Gulani A.C. (2025). StatPearls.

[4] Kahn H.A., Leibowitz H.M., Ganley J.P. (1977). The Framingham Eye Study. I. Outline and major prevalence findings. Am J Epidemiol.

[5] Klein B.E., Howard K.P., Lee K.E. (2012). The relationship of cataract and cataract extraction to age-related macular degeneration: the Beaver Dam Eye Study. Ophthalmology.

[6] Joachim N., Mitchell P., Burlutsky G. (2015). The incidence and progression of age-related macular degeneration over 15 years: the Blue Mountains Eye Study. Ophthalmology.

[7] Hofman A., Breteler M.M., van Duijn C.M. (2007). The Rotterdam Study: objectives and design update. Eur J Epidemiol.

[8] Bhuiyan A., Wong T.Y., Ting D.S.W. (2020). Artificial intelligence to stratify severity of Age-Related Macular Degeneration (AMD) and predict risk of progression to late AMD. Transl Vis Sci Technol.

[9] Burlina P.M., Joshi N., Pacheco K.D. (2018). Use of deep learning for detailed severity characterization and estimation of 5-year risk among patients with age-related macular degeneration. JAMA Ophthalmol.

[10] Guymer R.H., Wu Z., Gao S. (2023). HONU: a multicenter, prospective, observational study of the progression of intermediate age-related macular degeneration. Invest Ophthalmol Vis Sci.

[11] Terheyden J.H., Dunbar H.M.P., Schmitz-Valckenberg S. (2025). Validating candidate endpoints for intermediate age-related macular degeneration trials in a multi-centre setting-lessons from the MACUSTAR Study. Eye (Lond).

[12] Age-Related Eye Disease Study Research Group (2000). Risk factors associated with age-related macular degeneration. A case-control study in the age-related eye disease study: Age-Related Eye Disease Study Report Number 3. Ophthalmology.

[13] Age-Related Eye Disease Study Research Group (2001). The age-related eye disease study (AREDS) system for classifying cataracts from photographs: AREDS Report No. 4. Am J Ophthalmol.

[14] Age-Related Eye Disease Study Research Group (2001). A randomized, placebo-controlled, clinical trial of high-dose supplementation with vitamins C and E, beta carotene, and zinc for age-related macular degeneration and vision loss: AREDS Report No. 8. Arch Ophthalmol.

[15] Age-Related Eye Disease Study Research Group (2001). A randomized, placebo-controlled, clinical trial of high-dose supplementation with vitamins C and E and beta carotene for age-related cataract and vision loss: AREDS Report No. 9. Arch Ophthalmol.

[16] Age-Related Eye Disease Study Research Group (2005). The age-related eye disease Study severity scale for age-related macular degeneration: AREDS Report No. 17. Arch Ophthalmol.

[17] Ferris F.L., Davis M.D., Clemons T.E. (2005). A simplified severity scale for age-related macular degeneration: AREDS Report No. 18. Arch Ophthalmol.

[18] Age-Related Eye Disease Study Research Group (1999). The Age-Related Eye Disease Study (AREDS): design implications. AREDS Report No. 1. Control Clin Trials.

[19] SanGiovanni J.P., Chew E.Y., Agrón E. (2008). The relationship of dietary ω-3 long-chain polyunsaturated fatty acid intake with incident age-related macular degeneration: AREDS Report No. 23. Arch Ophthalmol.

[20] Fritsche L.G., Igl W., Bailey J.N. (2016). A large genome-wide association study of age-related macular degeneration highlights contributions of rare and common variants. Nat Genet.

[21] Likert R. (1932). A technique for the measurement of attitudes. Arch Psychol.

[22] Age-Related Eye Disease Study Research Group (2001). The Age-Related Eye Disease Study system for classifying age-related macular degeneration from stereoscopic color fundus photographs: the Age-Related Eye Disease Study Report Number 6. Am J Ophthalmol.

[23] Age-Related Eye Disease Study Research Group (2012). database of Genotypes and Phenotypes.

[24] Merle B.M., Silver R.E., Rosner B., Seddon J.M. (2015). Adherence to a Mediterranean diet, genetic susceptibility, and progression to advanced macular degeneration: a prospective cohort study. Am J Clin Nutr.

[25] Toulouie S., Chang S., Pan J. (2022). Relationship of retinal vessel caliber with age-related macular degeneration. J Ophthalmol.

[26] Seddon J.M., Reynolds R., Maller J. (2009). Prediction model for prevalence and incidence of advanced age-related macular degeneration based on genetic, demographic, and environmental variables. Invest Ophthalmol Vis Sci.

[27] Fritsche L.G., Chen W., Schu M. (2013). Seven new loci associated with age-related macular degeneration. Nat Genet.

[28] Klein R., Chou C.-F., Klein B.E.K. (2011). Prevalence of age-related macular degeneration in the US population. Arch Ophthalmol.

[29] Zambelli-Weiner A., Crews J.E., Friedman D.S. (2012). Disparities in adult vision health in the United States. Am J Ophthalmol.

[30] Zhang X., Cotch M.F., Ryskulova A. (2012). Vision health disparities in the United States by race/ethnicity, education, and economic status: findings from two nationally representative surveys. Am J Ophthalmol.

