# Supplementary material for: A Datasheet for the Age-Related Eye Disease Study (AREDS) on the database of Genotypes and Phenotypes
Source: Ophthalmol Sci. 2026 Feb 11;6(4):101115. doi: 10.1016/j.xops.2026.101115 (PMC12997207; doi:10.1016/j.xops.2026.101115)
Supplement: Appendix A [file mmc1.pdf]

APPENDIX A: Participant eligibility criteria

General Eligibility Criteria:

1.Randomized within 4 months following the Qualifying Visit (Chapter 6).

2. Age criteria:
    1. AMD Category 1 and 2 participants: Age 60 through 80 years at the Qualifying Visit.
    2. AMD Category 3 or 4 participants: Age 55 through 80 years at the Qualifying Visit.
    3. Participants 78 years of age at the time of Phase I registration are eligible regardless of age at the Qualifying Visit.

3. Stop taking any vitamins, minerals, or other supplements containing vitamin C, vitamin E, beta-carotene, zinc, or copper, other than those supplied by AREDS. Participants must have demonstrated that they have taken at least 75 percent of their run-in medication and have indicated that they are willing to take the AREDS Study Medication for the next 7 years.

4. Likely to be available, willing, and able to undergo examinations at 6-month intervals for at least 7 years.

5. Sign two informed consent forms

Ocular Eligibility Criteria:

1. Visual acuity score at the Qualifying Visit for
    1. AMD Category 1 or 2: ≥74 letters in each eye
    2. AMD Category 3: ≥ 74 letters in at least one eye with large drusen or geographic atrophy not involving the center of the macula.
    3. AMD Category 4 participants with advanced AMD in one eye: ≥ 74 letters in the eye without advanced AMD.
    4. AMD Category 4 participants without advanced AMD in either eye: ≥ 74 or more in one eye, ≤73 or less in the fellow eye.

2. Media clarity
    1. AMD Categories 1 and 2: sufficiently clear to discern potential small (< 63 microns in diameter) punctate or hard drusen located within a 2 disc diameter radius of the center of the macula.
    2. AMD Categories 3 and 4: sufficiently clear to allow photographic assessment of large drusen and discernment of advanced AMD in early stages.

3.Pupillary dilation ≥5 mm bilaterally. Dilation <5 mm in an aphakic eye in a participant with AMD Category 3 or 4 is eligible with adequate quality fundus photographs.
